# Cost-Effectiveness of [^18^F] Fluoroethyl-L-Tyrosine for Temozolomide Therapy Assessment in Patients With Glioblastoma

**DOI:** 10.3389/fonc.2019.00814

**Published:** 2019-08-28

**Authors:** Tristan Baguet, Jeroen Verhoeven, Filip De Vos, Ingeborg Goethals

**Affiliations:** ^1^Laboratory of Radiopharmacy, Ghent University, Ghent, Belgium; ^2^Department of Nuclear Medicine, University Hospital Ghent, Ghent, Belgium

**Keywords:** [^18^F] fluoroethyl-L-tyrosine, cost-effectiveness, positron emission tomography, glioblastoma, temozolomide

## Abstract

**Background and Purpose:** Glioblastomas are the most aggressive of all gliomas. The prognosis of these gliomas, which are classified as grade IV tumors by the World Health Organization (WHO), is poor. Combination therapy, including surgery, radiotherapy, and chemotherapy has variable outcomes and is expensive. In light of rising healthcare costs, there are societal demands for the justification of medical expenses. Therefore, we calculated the cost-effectiveness of follow-up [^18^F] fluoroethyl-L-tyrosine ([^18^F] FET) positron emission tomography (PET) scans performed on patients with glioblastoma after surgery and before commencing temozolomide maintenance treatment.

**Materials and Methods:** To determine the cost-effectiveness of follow-up [^18^F] FET PET procedures, we examined published clinical data and calculated the associated costs in the context of Belgian healthcare. We subsequently performed one-way deterministic sensitivity analysis and Monte Carlo analysis on the calculated ratios.

**Results:** The decision tree based on overall survival rates showed that the number of non-responders identified using PET was 57.14% higher than the number of non-responders identified using conventional MRI. Further, the decision tree based on progression-free survival rates revealed a comparable increase of 57.50% non-responders identified. The calculated cost of two required PET scans per patient during the follow-up treatment phase was 780.50 euros. Two cost-effectiveness ratios were determined for overall survival and progression-free survival rates. Both of these calculations yielded very similar results: incremental cost-effectiveness ratios of 1,365.86 and 1,357.38 euros, respectively, for each identified non-responder. The findings of the sensitivity analysis supported the calculated results, confirming that the obtained data were robust.

**Conclusion:** Our comparative study of conventional MRI and [^18^F] FET PET revealed that the latter is a valuable tool for predicting the treatment responses of patients with glioblastomas to follow-up temozolomide maintenance treatment while considering its cost-effectiveness. Thus, [^18^F] FET PET scans enable clinical outcomes to be predicted accurately and at a low cost. Moreover, given the robustness of the data in the sensitivity analyses, the level of certainty of this outcome is acceptable.

## Introduction

Glioblastomas account for 54% of all gliomas in adults and are considered the most aggressive tumors within this category (1). Categorized by the World Health Organization (WHO) as grade IV gliomas, they either develop from a low-grade glioma or originate *de novo*. Symptoms are usually severe and are caused by space-occupying lesions. The state-of-the-art treatment consists of surgery followed by adjuvant combination therapy that entails radiochemotherapy and six cycles of maintenance chemotherapy using temozolomide (TMZ) (2, 3). However, the prognosis remains poor, with only one-third of patients surviving for more than 2 years after diagnosis (4, 5).

In clinical practice, symptomatic patients with a suspected brain tumor undergo magnetic resonance imaging (MRI) of the brain. MRI is the imaging technique of choice for diagnosis because of its excellent soft tissue contrast (6, 7). However, there are shortcomings associated with this technique. In particular, during the follow-up phase, it is difficult to discriminate between pseudo-progression after the completion of treatment vs. tumor progression using conventional MRI. This is because contrast enhancement with MRI is an aspecific finding that merely indicates leakage of the contrast medium through the blood-brain-barrier, which may be treatment related (8, 9).

Advanced MR imaging modalities, such as diffusion- and perfusion-weighted MRI and MR spectroscopy, are potentially able to differentiate between pseudo- and real progression more effectively than anatomical MRI used on its own can. Moreover, positron emission tomography (PET) imaging may enable this differentiation, as it allows for the visualization of specific biological processes. For brain tumor imaging conducted with PET, amino acid analogs are preferred as PET tracers. This is because they do not show significant uptake in inflammatory tissue and are therefore less influenced by phenomena such as pseudoprogression and pseudoresponse (8, 9).

It is also noteworthy that after the treatment is completed, biological changes usually precede anatomical alterations. Therefore, PET imaging can potentially enable early detection of therapy responses (10, 11).

This role of PET imaging was further explored by Galldiks et al. (12), who compared the capability of [^18^F]-fluoroethyl-L-tyrosine ([^18^F] FET) PET for assessing therapy responses after administering TMZ and radiotherapy with that of MRI. The authors correlated changes in [^18^F] FET uptake with changes in contrast-enhancing tumor volume shown in T1-weighed MRI images after surgery and treatment with TMZ. They concluded that [^18^F] FET PET could be a valuable tool for predicting the therapy outcome prior to the commencement of TMZ maintenance therapy. This is an important finding given the potentially severe side-effects of TMZ. Hence, the authors argued that therapy should be tailored to each specific patient based on the results of the acquired [^18^F] FET PET image.

However, incorporating [^18^F] FET PET into daily clinical routines is associated with increased medical costs and can only be justified when there are sufficient benefits associated with its use in terms of improved survival rates as well as quality of life. Therefore, our aim was to perform an economic analysis of [^18^F] FET PET imaging in patients with glioblastoma during follow-up treatment. The economic analysis was done on a sample of glioblastoma patients to obtain a homogeneous group, receiving the same treatment. For every patient, therapy consisted of surgery followed by adjuvant radiotherapy and concomitant temozolomide. Upon completion of radiochemotherapy the patient received maintenance temozolomide. To the best of our knowledge, a comparison of the cost-effectiveness of [^18^F] FET PET and MRI for predicting therapy responses has not been undertaken to date.

## Materials and Methods

### Input Data

The effectiveness of identifying responders and non-responders with PET and MRI was determined using clinical data obtained from Galldiks et al. (12). Twenty-five patients diagnosed with glioblastoma, who underwent resection (13 partial resections and 12 gross total resections), were included in their study. Both PET and MRI scans were performed 11–20 days post-surgery (MRI-1, FET-1). Following neurosurgery, the patients underwent adjuvant combined radiotherapy and TMZ treatment. Within 7–10 days after radiochemotherapy commenced, PET and MRI scans were repeated (MRI-2, FET-2). Following a 4-weeks break, the patients underwent another six cycles of TMZ. A third set of MRI and PET scans (MRI-3, FET-3) was performed 6–8 weeks after the completion of radiochemotherapy. Drawings of three-dimensional contrast-enhancing volumes were used to delineate the tumors on the MRI images, whereas a 1.6 cut-off tumor-to-background (TBR) value was used to delineate tumor tissue in the [^18^F] FET images based on previous findings on the best cut-off value for distinguishing tumor tissue from non-tumor tissue (13). The authors found that changes in the Gd-volumes observed between MRI-1 and MRI-2 or MRI-3 scans did not have any predictive value. However, a decrease of at least 20% of the maximum tumor-to-background (TBR_max_) value between the FET-1 and FET-2 imaging enabled responders to be differentiated from non-responders. The patients' characteristics can be found in Table 1.

**Table 1:** Patients' characteristics: MRI and PET data for scans 1 and 2 conducted on each patient [Source: (12)].

	**MRI [Gd-volume (cm^3^)]**			**PET (TBR_max_)**			**OS (months)**		**PFS (months)**	
**Nr**.	**1**	**2**	**Status^a^**	**1**	**2**	**Status^b^**	**Months**	**Status^c^**	**Months**	**Status^d^**
1	0.9	4.3	0	2.6	/	/	16.1	1	14.1	1
2	14.2	22.0	0	4.1	4.0	0	9.3	0	8.3	1
3	/^e^	14.8	/	3.5	4.6	0	5.6	0	5.1	0
4	6.0	3.7	1	3.6	2.8	1	22.8	1	9.4	1
5	31.6	5.2	1	4.1	3.4	0	6.9	0	5.4	0
6	0.6	2.7	0	3.6	3.0	0	28.5	1	7.2	1
7	6.1	0.5	1	4.6	3.2	1	28.5	1	19.3	1
8	0.0	0.0	1	2.0	1.7	0	14.8	1	13.9	1
9	1.3	6.4	0	2.3	3.2	0	9.3	0	4.7	0
10	1.1	0.6	1	3.7	2.3	1	16.1	1	5.3	0
11	16.8	8.7	1	3.3	3.3	0	2.8	0	2.8	0
12	1.6	0.9	1	2.2	1.5	1	28.7	1	28.7	1
13	5.2	/	/	2.0	2.1	0	8.5	0	5.5	0
14	19.4	2.3	1	4.9	2.8	1	10.5	1	5.2	0
15	6.8	/	/	3.6	2.5	1	14.8	1	10.3	1
16	19.5	3.9	1	4.8	3.8	1	14.3	1	3.3	0
17	8.2	0.4	1	3.3	2.6	1	15.4	1	12.9	1
18	0.8	6.2	0	3.1	2.5	0	9.8	0	7.8	1
19	1.3	6.9	0	3.8	2.4	1	20.9	1	9.3	1
20	2.2	0.6	1	3.1	2.5	0	8.2	0	6.6	1
21	4.9	4.6	1	2.9	2.6	0	9.9	0	5.8	0
22	3.7	2.8	1	2.4	2.8	0	13.8	1	3.8	0
23	0.1	1.8	0	2.4	2.0	0	15.7	1	15.7	1
24	6.5	/	/	2.5	/	/	6.8	0	5.2	0
25	6.4	0.0	1	2.0	1.4	1	13.3	1	13.3	1

a *Criteria for MRI scan: R, Responder (Gd vol 2 < 1); NR, Non-responder (Gd vol 2 > 1). Status 1 = R; Status 2 = NR*.

b *Criteria for PET scan: R, Responder (TBR_max_ 2 < 80% TBR_max_ 1); NR, Non-responder (TBR_max_ 2 > 80% TBR_max_ 1). Status 1 = R; Status 2 = NR*.

c *Criteria for OS scan: R, Responder (OS > 10 months); NR, Non-responder (OS < 10 months). Status 1 = R; status 2 = NR*.

d *Criteria for PFS scan: R, Responder (PFS > 6 months); NR, Non-responder (PFS < 6 months). Status 1 = R; status 2 = NR*.

e */, data not available*.

## Constructing the Decision Tree Model

**Figure 1:**
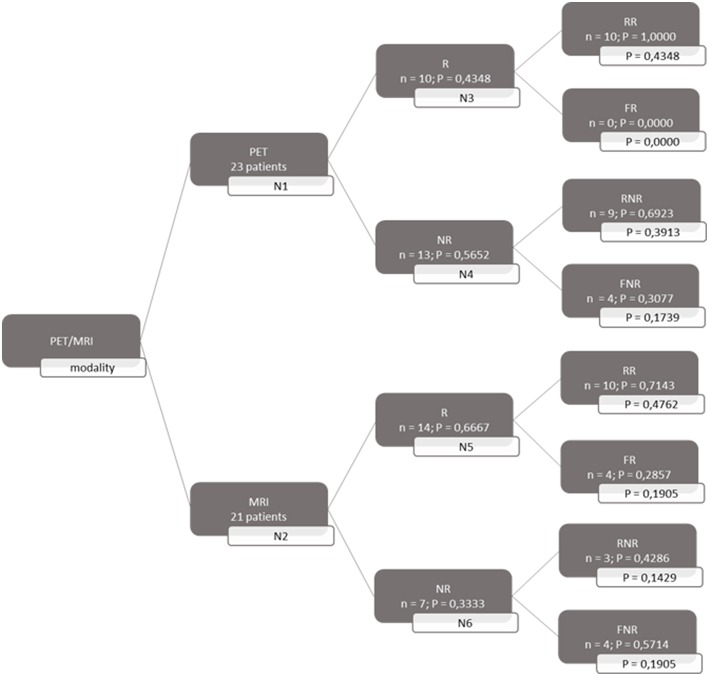
Decision tree based on overall survival patients. 23 patients were available for PET analysis and 21 for MRI analysis. N1 & N2 gave chance node to be responder (R) for, respectively, PET and MRI. Chance nodes N3 & N5 gave the chance to be a real responder (RR) with PET and MRI. N4 and N6 gave the chance to be real non-responder (RNR) for PET and MRI. Non-responder (NR); non-real responder (NRR); and non-real non-responder (NRNR) are equal to 1 minus the chance to be R; RR and RNR. N = number patients. P in most right transparent framework gives the total chance to this event (is calculated by multiplying the previous two chance nodes).

**Figure 2:**
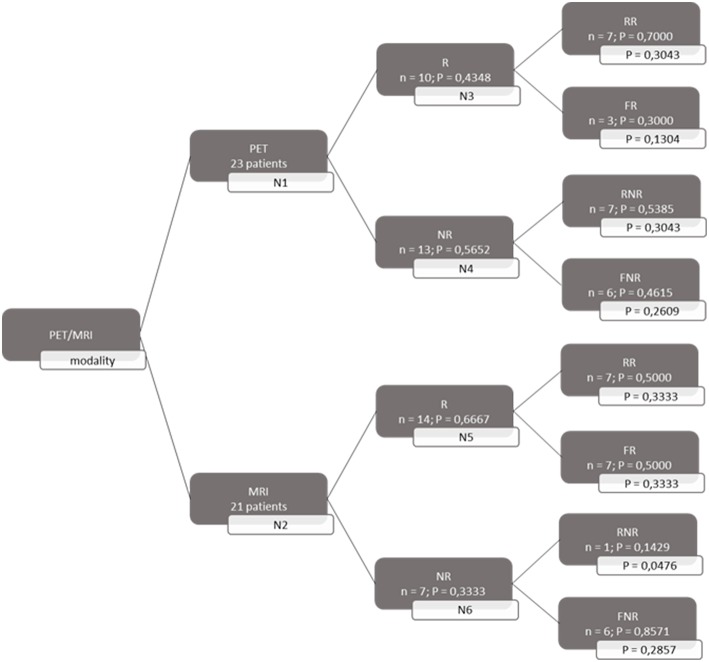
Decision tree based on progression free survival patients. 23 patients were available for PET analysis and 21 for MRI analysis. N1 & N2 gave chance node to be responder (R) for, respectively, PET and MRI. Chance nodes N3 & N5 gave the chance to be a real responder (RR) with PET and MRI. N4 and N6 gave the chance to be real non-responder (RNR) for PET and MRI. Non-responder (NR); non-real responder (NRR); and non-real non-responder (NRNR) are equal to 1 minus the chance to be R; RR and RNR. N = number patients. P in most right transparent framework gives the total chance to this event (is calculated by multiplying the previous two chance nodes).

A decision tree was developed using the Microsoft Office Word program (Microsoft Office, version 1803) to compare the clinical effectiveness of PET and MRI. This model was derived from previously constructed decision trees reported in the literature (14, 15). To develop the decision tree, the patients who featured in the study by Galldiks et al. (12) were divided into two categories: responders and non-responders according to differences found in the characteristics of the images generated during the first and subsequent PET and MRI scans (Figures 1, 2). Chance node one (N1) indicated the possibility of a patient being a responder according to [^18^F] FET PET features. The patient was defined as a responder when there was a 20% decrease in the TBR_max_ value obtained for FET-2 compared with the value obtained for FET-1. Chance node two (N2) indicated the possibility of a patient being a responder according to MR features defined as no change or a decrease in the contrast-enhancing volume for MRI-2. In chance node three (N3), the PET-responders were divided into true responders and false responders on the basis of clinical endpoints. Patients who were both clinical and PET responders were defined as true responders. Those who were PET responders, but not clinical responders were categorized as false responders. Patients identified as non-responders on the basis of the PET images were similarly assigned as true non-responders and false non-responders in chance node four (N4). In chance node five (N5), true responders were separated from false responders according to their clinical endpoints and differential changes in contrast-enhancing volumes. Chance node six (N6) showed true non-responders and false non-responders, as identified from MRI scans. A decision tree was developed for each of the two clinical endpoints used. In the first decision tree, patients with an overall survival rate of more than 10 months were considered as clinical responders. This definition is arbitrarily based on the median overall survival rate derived from Johnson et al. (16). In the second decision tree, a clinical responder was defined as one with a progression-free survival rate of 6 months or more. A progression-free survival rate of 6 months has already been shown to be the best indicator for differentiating between long-term and short-term survivors (17, 18).

Because the prognosis after a glioblastoma diagnosis is poor, it is of utmost importance to identify patients who are not showing an early response to therapy. Accordingly, TMZ maintenance therapy can be omitted, thereby avoiding possible severe side effects, and a second line treatment can be proposed. Hence, we calculated the probability of being able to identify a non-responder.

### Cost Calculation

The costs associated with diagnosis using [^18^F] FET PET were determined in the context of Belgian healthcare. The costs included in the calculation were the reimbursement of expenses incurred for services relating to the use of [^18^F] FET PET by the National Institute for Health and Disability Insurance, which is the federal public body responsible for social security provision in Belgium. The cost-effectiveness of [^18^F] FET PET was expressed as an incremental cost-effectiveness ratio (ICER).

There are two cost centers relating to [^18^F] FET PET imaging at the Ghent University Hospital. The first entails government incentives for the purchase and maintenance of PET scanners. The second cost center entails reimbursement of expenses incurred by the hospital through the provision of services relating to the acquisition and interpretation of PET scans by the National Institute for Health and Disability Insurance.

### Cost-Effectiveness

The cost-effectiveness of [^18^F] FET PET used to assess therapy responses to TMZ of patients with glioblastoma was expressed as the ICER for each identified non-responder as follows:

$$\begin{array}{l} ICER=\frac{Cost\;\left[18F\right]FET\;}{Effectiveness\;\left[18F\right]FET-Effectiveness\;MRI} \end{array}$$

### Sensitivity Analysis

To determine the impact of each independent variable on the cost calculation, a one-way deterministic sensitivity analysis was performed. We used confidence intervals derived from a similar study (14), as shown in Table 2, to estimate the degree of uncertainty regarding the parameters of the decision tree. We also determined variability relating to the number of scans performed annually, which influences the cost of [^18^F] FET PET. The calculated results were plotted in a tornado diagram (see Table 3).

**Table 2:** Chance node intervals for decision tree 1 based on the overall survival rate and for decision tree 2 based on the progression-free survival rate developed for the one-way deterministic sensitivity analysis.

**Chance node**	**Interval decision tree 1 (Chance=)**		**Interval decision tree 2 (Chance=)**	
N1	0.2848	0.5848	0.2848	0.5848
N2	0.5167	0.8167	0.5167	0.8167
N3	0.9250	1.0000	0.6250	0.7750
N4	0.6173	0.7673	0.4635	0.6135
N5	0.6393	0.7893	0.4250	0.5750
N6	0.3536	0.5036	0.0679	0.2179
# scans	5,062	6,189	5,062	6,189

*An interval of 30 percent points was chosen for chance nodes one and two while an interval of 15 percent points was chosen for chance nodes three and four*.

**Table 3:** Input variables used in a Monte Carlo analysis.

	**Decision tree 1**		**Decision tree 2**	
**Variable**	**Calculated value**	**Standard deviation**	**Calculated value**	**Standard deviation**
# scans	5,626	287	5,626	287
N1	0.4348	0.0750	0.4348	0.0750
N2	0.6667	0.0750	0.6667	0.0750
N3	1.0000	0.0375	0.7000	0.0375
N4	0.6923	0.0375	0.5385	0.0375
N5	0.7143	0.0375	0.5000	0.0375
N6	0.4286	0.0375	0.1429	0.0375

*Calculated values and their standard deviations are shown for each variable applied in the cost-effectiveness analysis*.

A Monte Carlo simulation was performed to analyze the probabilistic sensitivity. For each independent variable, 10,000 *at random* values within the normal distribution were chosen. These values were used to calculate new ICER values. All of the simulations were performed using the Microsoft Office Excel program (Microsoft Office, version 1803).

## Results

### Effectiveness

Both decision trees showed that the use of [^18^F] FET PET imaging increased the number of identified non-responders. The first decision tree constructed with the overall survival rate showed that the number of non-responders identified using PET imaging (100%) was 57.14% more than the number of non-responders identified with MRI imaging (42.86%). The second decision tree constructed with the progression-free survival revealed a comparable increase (57.5%) in the identification of non-responders with PET (70%) compared to MRI (12.5%).

### Cost Calculation

The total cost calculated for the follow-up phase for each patient was 780.50 euros (see Supplementary Material). The resulting ICER values calculated for the first and second decision tree were 1,365.86 and 1,357.38 euros, respectively for each non-responder identified on PET but not on MRI.

### Sensitivity Analysis

**Figure 3:**
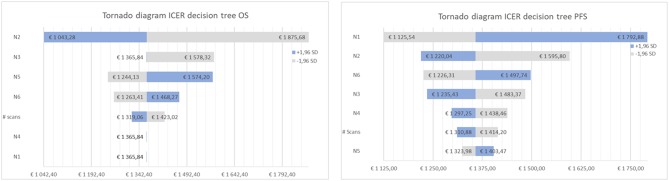
Tornado diagram of the cost-effectiveness ratio for decision tree one (left), based on overall survival (OS) and two (right), based on progression free survival (PFS).

Tables 4, 5 present the results of the one-way sensitivity analysis, and Figure 3 depicts the tornado diagrams. The minimum and maximum ICER values were 1,043.28 and 1,875.68 euros, respectively, for the first decision tree and 1,125.54 and 1,792.88 euros, respectively, for the second decision tree. The minimum and maximum costs of a follow-up [^18^F] FET PET session were 613.74 and 813.20 euros, respectively. N1 and N2 showed the greatest impacts relating the minimum and maximum ICER values, which were direct outcomes of the wider variability.

**Table 4:** Effect of the number of scans performed annually on the cost of [^18^F] FET-based follow-up therapy.

	**Interval cost [^18^F] fluoroethyl-L-tyrosine scan**	
	**Lower limit interval**	**Upper limit interval**
Number of scans	5,063	6,189
Cost [^18^F] fluoroethyl-L-tyrosine	813.20 euros	735.74 euros

**Table 5:** Data used for decision tree 1 (overall survival rate) and decision tree 2 (progression-free survival rate) in the one-way deterministic sensitivity analysis.

**Chance node**	**Interval decision tree 1 (chance** **=)**		**Interval decision tree 2 (chance** **=)**	
N1	0.2848	0.5848	0.2848	0.5848
P event	0.5714	0.5714	0.6934	0.4353
N2	0.5167	0.8167	0.5167	0.8167
P event	0.4161	0.7481	0.4891	0.6397
N3	0.9250	1.0000	0.6250	0.7750
P event	0.4945	0.5714	0.5262	0.6318
N4	0.6173	0.7673	0.4635	0.6135
P event	0.5714	0.5714	0.5426	0.6017
N5	0.6393	0.7893	0.4250	0.5750
P event	0.6273	0.4958	0.5895	0.5561
N6	0.3536	0.5036	0.0679	0.2179
P event	0.6178	0.5316	0.6365	0.5211

*A change in effectiveness was observed for every change in a chance node*.

**Figure 4:**
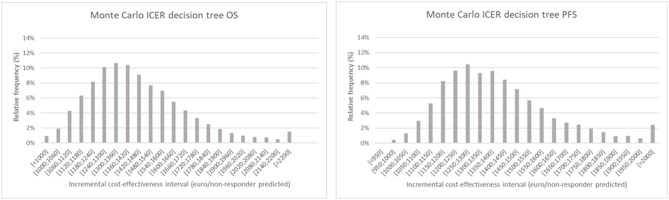
Monte Carlo simulation of the incremental cost-effectiveness ratio for decision tree one (left), based on overall survival and two (right), based on progression free survival. The chart shows the relative frequency of the probability on a certain incremental cost-effectiveness.

The results of the Monte Carlo analysis, depicted in Figure 4, showed a narrow distribution of the ICER values around the average. This finding implies high levels of data robustness and of the reliability of the calculated values (see Tables 6, 7).

**Table 6:** Data obtained from the Monte Carlo analysis conducted for decision tree 1.

	**Cost FET (€)**	**P Corr NR**	**ICER (€)**
Average	781.2	0.5582	1,444
Maximum	851.2	0.9207	3,531
Minimum	726.8	0.2242	837
Standard deviation	15.2	0.0957	273.5

*Average, maximum values, minimum values, and their standard deviations, were obtained for the following parameters: cost of [^18^F] fluoroethyl-L-tyrosine study (cost FET), possibility of identifying non-responders (P corr NR, chance correct non-responder), and the incremental cost-effectiveness ratio (ICER)*.

**Table 7:** Data obtained from the Monte Carlo analysis conducted for decision tree 2.

	**Cost FET (€)**	**P Corr NR**	**ICER (€)**
Average	781.1	0.5704	1,405
Maximum	846.2	0.8208	3,083
Minimum	733.4	0.2458	948
Standard deviation	15.3	0.0862	241.8

*The average, maximum, and minimum values, and their standard deviations, were obtained for the following parameters: cost of [^18^F] fluoroethyl-L-tyrosine study (cost FET), possibility of recognizing non-responders (P corr NR, chance correct non-responder), and the incremental cost-effectiveness ratio (ICER)*.

## Discussion

Given limited resources available for health care, it is essential to ensure optimally efficient allocation of financial resources. With the development of new diagnostic imaging modalities, clinicians can tailor the therapy for individual patients. However, most imaging techniques require substantial investments and should ideally be used only when there are indications that the added value outweighs the cost. In this context, the cost-effectiveness of [^18^F] FET PET for assessing the therapy response of patients with glioblastoma to TMZ treatment was investigated. Our data indicated that the identification of non-responders at a low incremental cost was feasible. In addition, the results of the sensitivity analysis revealed that these calculations were robust, implying that there was a low level of variability.

The use of PET as a prognostic tool for high-grade gliomas has been investigated over a period of several years. A previous study conducted on [^18^F] dihdryoxyphenylalanine ([^18^F] FDOPA) within a large target population did not find that the amino acid PET tracer had any prognostic power (19). However, studies on [^18^F] FET have shown that this tracer could have prognostic potential (12, 13). These conflicting results can be attributed to differences in target populations or in PET tracers. The results of our decision tree model indicate that [^18^F] FET can enable the differentiation of long-term survival and short-term survival. Three studies conducted by Heinzel et al. have confirmed the cost-effectiveness of the [^18^F] FET PET modality for patients with glioblastoma (14, 15, 20). In the first study, the authors calculated the cost-effectiveness of [^18^F] FET PET-guided biopsy for diagnosing gliomas (20). The second study investigated the cost-effectiveness of managing [^18^F] FET PET-based therapy using bevacizumab and irinotecan (14). The third study examined the cost-effectiveness of [^18^F] FET PET in the evaluation of recurrent metastasis in the brain (15). The ICER values reported in the three papers varied between 2,821 and 9,114 euros per diagnosis, depending on indications and scenarios. In our study, we investigated the cost-effectiveness of [^18^F] FET PET used in TMZ therapy management. Our results showed an ICER value for each identified non-responder. However, comparison of our results to the literature was difficult because ICER values were expressed per non-responder instead of per diagnosis. Therefore, we provided additional ICER values of 3,769.77 euros and 2,427.07 euros per diagnosis obtained, respectively, for overall survival and progression-free survival rates in the section with Supplemental Information. These values are in line with the ICER values reported in the above-mentioned studies by Heinzel et al.

In light of our results, we recommend the use of [^18^F] FET PET scans during the follow-up treatment phase for patients with glioblastoma. The use of this diagnostic modality enables the identification of non-responders at an early stage and at an acceptable cost. Moreover, the avoidance of ineffective TMZ maintenance therapy in non-responders is cost-effective and results in considerable cost-saving.

The data described here merely represent a starting point for further research. It should also be noted that there were several shortcomings in our study. Only 25 patients were included in the study conducted by Galldiks et al. (12) that constituted the basis for our construction of the decision trees in this study. This limited sample of patients led to increased variability in the clinical data.

The differentiation of clinical responders and non-responders in this study was based on both the overall survival and progression-free survival rates. In the first decision tree, the criterion used to define a clinical responder was an overall survival rate of a minimum of 10 months. This criterion was derived from the median overall survival rate of patients diagnosed with glioblastoma, which was 10 months (16). However, it is important to note that the median overall survival rate identifies the overall survival of 50% of the patient population and does not provide any information relating to long-term and short-term survival. Consequently, a second decision tree was developed, using the criterion of a progression-free survival rate of 6 months to divide the patients into clinical responders and non-responders. Although a progression-free survival rate of 6 months has already been shown to be valid for distinguishing long-term survivors from short-term survivors, it is not a primary endpoint in clinical trials. Because both overall survival and progression-free survival rates each have their merits, two decision trees were constructed. Notably, ICER values obtained for both decision trees were very similar.

Galldiks et al. (12) conducted a comparative study of PET and MRI. It is important to note that in clinical practice, a PET scan will generally be performed alongside an MRI scan for patients with glioblastoma. Therefore, the cost of the MRI scan was not subtracted from that of the [^18^F] FET PET scan in the ICER calculations. Consequently, a comparison of the cost of MRI with the combined cost of PET and MRI would also be appropriate. It is possible that combining both image modalities could lead synergistically to greater accuracy in terms of specificity and/or sensitivity than through their individual use. Advanced MR sequences also provide metabolic information to some extent. Therefore, it would be of great interest to do a comparing cost-effectiveness study between advanced MR sequences and PET to see which modality is the most cost-effective.

Therapy-related effects on imaging cannot be excluded. The findings of Chiaravalloti et al. suggest that radiotherapy has a long-term influence on the results of PET scans (21). However, an effective intervention entailing a follow-up PET scan in maintenance therapy necessitates its performance shortly after radiochemotherapy has been completed.

## Conclusion

[^18^F] FET PET is a valuable tool that enables the treatment outcome to be predicted prior to commencing maintenance TMZ therapy in patients with glioblastoma. It is, therefore, more cost-effective than conventional MRI. Specifically, it enables the clinical outcome to be predicated accurately and at a low cost. In addition, the results of our sensitivity analyses indicated that this outcome could be achieved with an acceptable level of certainty given the robustness of the data.

## Data Availability

The datasets generated/analyzed can be found here: doi: 10.2967/jnumed.111.098590. The link to the full text is: http://jnm.snmjournals.org/content/53/7/1048.long.

## Author Contributions

TB, JV, FD, and IG: conceptualization, writing—review, and editing. TB and JV: data curation, formal analysis, investigation, software, visualization, and validation. TB, JV, and IG: methodology. FD and IG: supervision, project administration, and resources. TB and IG: writing—original draft.

### Conflict of Interest Statement

The authors declare that the research was conducted in the absence of any commercial or financial relationships that could be construed as a potential conflict of interest.

## Abbreviations

[^18^F] FET: [^18^F] Fluoroethyl-L-tyrosine
[^18^F] FDOPA: [^18^F] fluorodihydroxyphenylalanine
ICER: Incremental cost-effectiveness ratio
MRI: Magnetic Resonance Imaging
N: Chance node
PET: Positron Emission Tomography
TBR: Tumor-to-background
TBRmax: maximal tumor-to-background
TMZ: Temozolomide
WHO: World Health Organization.
