# Supplementary material for: Cost-Effectiveness of [18F] Fluoroethyl-L-Tyrosine for Temozolomide Therapy Assessment in Patients With Glioblastoma
Source: Front Oncol. 2019 Aug 28;9:814. doi: 10.3389/fonc.2019.00814 (PMC6722181; doi:10.3389/fonc.2019.00814)
Supplement: Supplementary file 1 [file Table_1.docx]

Supplementary Material

# Cost calculation

The cost centers included were the expenses for [^18^F]FET scans paid by the National Institute for Health and Disability Insurance (NIHDI) and the government necessary to conduct the diagnosis. A [^18^F]FET scan applied for in the University Hospital of Ghent includes two cost centers for the community. Firstly, the yearly grants given by both the Flemish and Belgian government for the PET-scanners. Secondly, the payments by the National Institute for Health and Disability Insurance to the University Hospital for the execution and interpretation of the PET-scan. These costs include the total cost of a [^18^F]FET PET-scan.

## Number of scans

To calculate the total cost per [^18^F]FET-scan it is important to know how many scans are performed each year per PET-camera as the Belgian and Flemish grants are fixed amounts per PET scanner. An increase in PET-scans per year results in a lower cost per scan. In addition, certain reimbursements by the NIHDI apply only for the first 3,400 scans. An increase in PET-scans here also results in a decreased cost per scan. In the period of 01/01/2017-01/10/2017 a total of 4,223 PET scans were performed in the University Hospital of Ghent. Extrapolation to 31/12/2017 results in a total of 5,626 scans for the year 2017. A standard deviation of 287 was calculated from the number of scans performed in the year 2015 (5,080), 2016 (5,506) and 2017 (5,626).

## Reimbursement [^18^F]FET tracer by National Institute for Health and Disability Insurance

A reimbursement of 60 euro for the [^18^F]FET tracer by the NIHDH is listed under: *Lijst van de vergoedbare radiofarmaceutische producten Hoofdstuk II < B Lijst van de therapeutische aangewende radiofarmaceutische producten < Paragraaf 10002 < F-18 (UZ GENT).* This document can be found on the website of the NIHDH (http://www.riziv.fgov.be/nl/themas/kost-terugbetaling/door-ziekenfonds/geneesmiddel-gezondheidsproduct/terugbetalen/radiopharma/Paginas/vergoedbare-radiofarmaceutische-referentielijsten-referentiebestanden.aspx#.Wsc2ZYhuZPY).

A second pharmaceutical cost of 1.72 euro for the 0.9% sodium chloride solution necessary for infusion is charged twice. The rates for these solutions can be found under the CNK-code 0725-010 on the website: http://ondpanon.riziv.fgov.be/SSPWebApplicationPublic/nl/Public/ProductSearch

## Hospital funding

Each year the Flemish hospitals receive financial resources from the Belgian government, Flemish government and NIHDH to meet their needs. Section B3 in the budget for financial resources contains the reimbursements hospitals receives for the performed PET-scans.

- The NIHDH provides two refunds for the execution of a PET-scan, on the one hand a reimbursement for section B3 of the budget for financial resources (50.49 euro) and on the other hand a PET-tracer reimbursement (107.55 euro). Both amounts are only refunded for the first 3,400 scans. The most recent circular letter stating the prices to the hospitals is: *Omzendbrief aan de ziekenhuizen 2017/04 Prijs: farmaceutische verstrekkingen: 01-06-2017.* http://www.riziv.fgov.be/nl/professionals/verzorgingsinstellingen/ziekenhuizen/Paginas/omzendbrieven-algemeen-ziekenhuis.aspx#.WsdA6IhuZPY.
- The Belgian government has a yearly contribution of 200,000 euro to the budget of financial resources which can be found under: *Nota bij de betekening van het budget van financiële middelen*. *< 2.De vaststelling van de verschillende onderdelen van het budget van financiële middelen op 1 juli 2016 < 2.3 Onderdelen A3 en B3 – Financiering NMR, radiotherapie en petscan*. *https://www.health.belgium.be/sites/default/files/uploads/fields/fpshealth_theme_file/nota_als_bijlage_aan_bfm_op_01072016.pdf.*
- The Flemish government donates an additional grant of 90,000 euro which can be found under *https://www.departementwvg.be/sites/default/files/media/Communicatie_toestelfinanciering.pdf.*

## Honorarium

In addition to the tracer and scanner related compensations the hospital also receives an honorarium linked to the performance of the nuclear physician. For a PET scan of head and neck-tumors the codes 442971-442982 apply. The codes and indications can be found under *"****§ 2.*** *Worden beschouwd als verstrekkingen die de bekwaming van geneesheer, specialist voor nucleaire geneeskunde (XN), vereisen :" =>* ***B. Tests of doseringen met gemerkte producten****: =>* ***d) quater.*** *PET-onderzoeken (Positron Emissie Tomografie) => 1. Onderzoek bij oncologische indicaties => Positronentomografisch onderzoek door coïncidentiedetectie met protocol en documenten, voor het geheel van het onderzoek, voor oncologische indicaties N 250. De verstrekking 442971-442982 is enkel aanrekenbaar in de volgende indicaties: => 2) in geval van een initieel uitbreidingsbilan van een maligne tumor: => g) van een hoofd- en halstumor.* This section can be found on the documents on website: http://www.riziv.fgov.be/nl/nomenclatuur/Paginas/default.aspx#.WuLQ3YhuZPY.

The reimbursement of 179.10 euro for code 442971-44298 is stated on *http://www.riziv.fgov.be/nl/themas/kost-terugbetaling/door-ziekenfonds/individuele-verzorging/honoraires/Paginas/arts-deel10.aspx#.WoBRsajiZPa*.

An additional honorarium of 0.65 euro for accredited physicians is provided by the NIHDH with the code 449912.

In the year 2017 an estimated number of 5,626 PET-scans were performed at the University Hospital of Ghent. Based on this number an average cost per PET-scan was calculated. The sum of 290,000 euro (90,000 from Flemish government and 200,000 euro from the Belgian government) equals to 51.55 euro grant per scan (= 290,000 euro/ 5,626 scans). Next, the segment of budget of financial resources is divided over the number of scans. The total budget was 537,336 euro ((50.49 euro +107.55 euro) *3 400 reimbursed examinations). Dividing 537,336 euro over 5,626 scans results in an amount of 95.51 euro per scan.

Adding up the different components results in a cost of 390.25 euro per scan. To determine the prognosis of a patient a total of two scans are necessary which brings the total cost to 780.50 euro per patient.

60 euro + 3.44 euro + 51.55 euro + 95.51 euro + 179.10 euro + 0.65 euro = 390.25 euro.

# Cost-effectiveness correct diagnosis

In addition to the cost-effectiveness to recognize one non-responder, the cost-effectiveness for one extra correct diagnosis was determined. [^18^F]FET showed an increase of 20.70% (61.90% with MRI vs 82.60% for PET) more correct prognoses in resemblance to MRI based on overall survival. A similar increase of 22.76% (38.12% with MRI vs 60.88% for PET) was seen based upon progression free survival. The incremental cost-effectiveness ratio for one correct prognosis was 3,769.77 euro based on overall survival and 3,427.07 euro based on progression free survival.

The one-way deterministic sensitivity analysis shows ICER intervals [3,082.91; 4,852.09] for decision tree based on overall survival and [2,774.44; 4,481.16] for the decision tree based on progression free survival. In the Monte Carlo analysis results are seen very similar to the Monte Carlo analysis of the ICER values for the recognition of a non-responder. Very robust data with a narrow distribution.

# Supplementary Figures and Tables

| 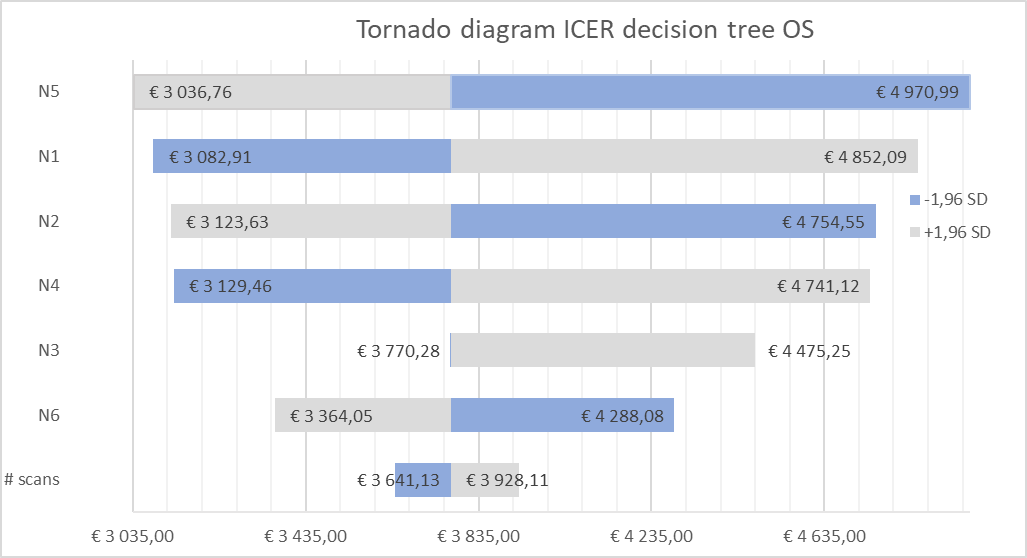 | 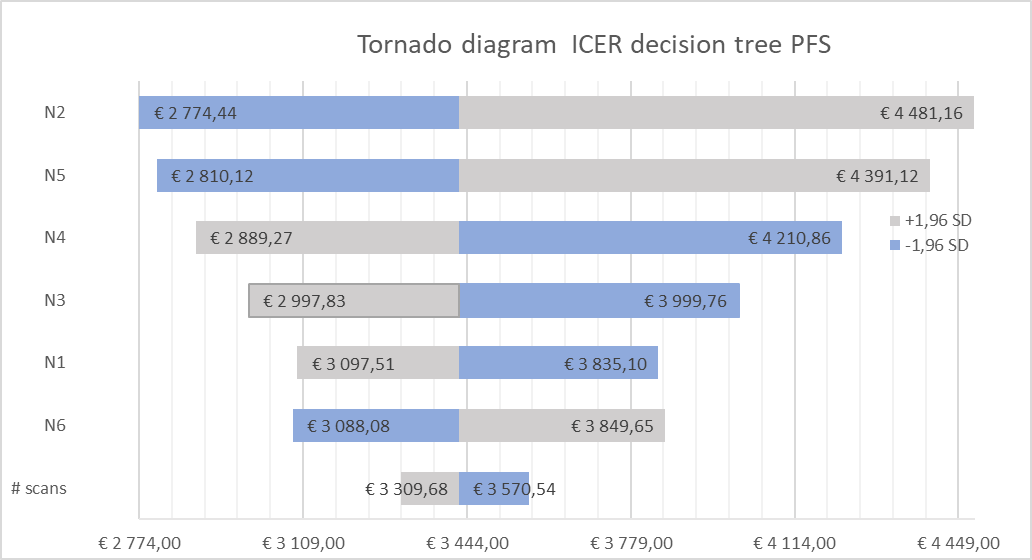 |
| --- | --- |

*Figure 1: Tornado diagram of the cost-effectiveness ratio for correct prognosis for decision tree based on overall survival (left) and progression free survival (right).*

| 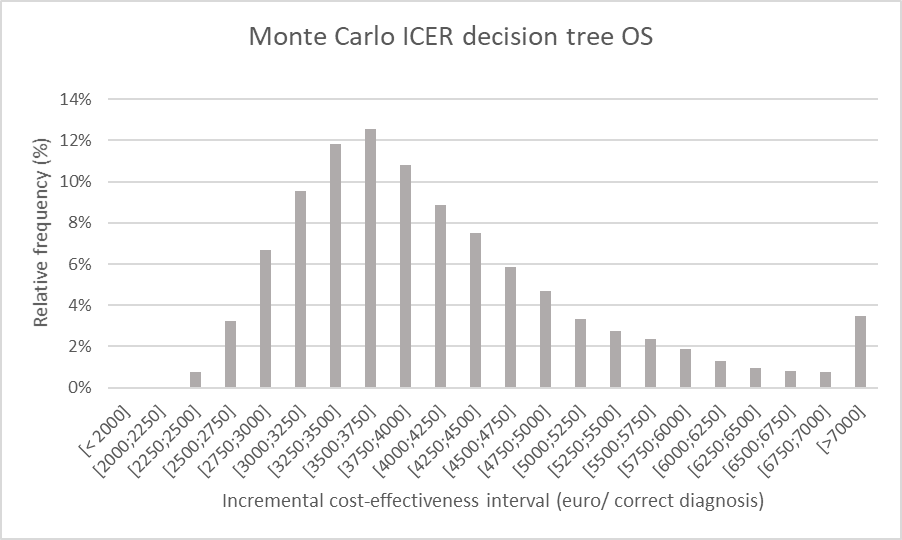 | 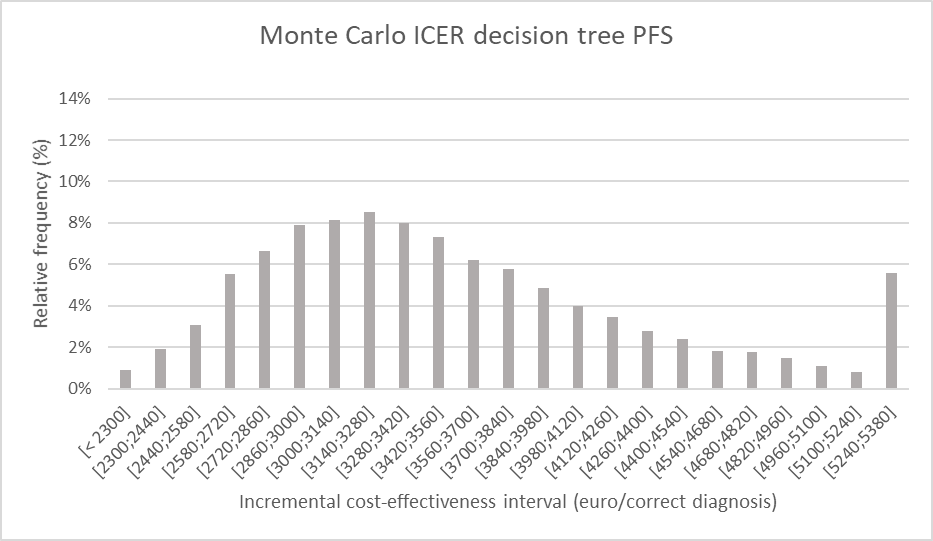 |
| --- | --- |

*Figure 2: Monte Carlo simulation of the incremental cost-effectiveness ratio for decision tree one (left), based on overall survival and two (right), based on progression free survival. The chart shows the relative frequency of the probability on a certain incremental cost-effectiveness*

*Table 1: Data Monte Carlo analysis for decision tree 1. It provides the average, maximum, minimum values and their standard deviation of the following parameters: cost of FET study, increase in chance of correct prognosis (P correct prognosis) and the ICER (incremental cost-effectiveness ratio).*

|  | Cost FET (€) | P correct prognosis | ICER (€) |
| --- | --- | --- | --- |
| Average | 781.2 | 0.2002 | 4,182 |
| Maximum | 851.2 | 0.3608 | 27,978 |
| Minimum | 726.8 | 0.0281 | 2,142 |
| Standard deviation | 15.2 | 0.0473 | 1,337 |

*Table 2: Data Monte Carlo analysis for decision tree 2. It provides the average, maximum, minimum values and their standard deviation of the following parameters: cost of FET study, increase in chance of correct prognosis (P correct prognosis) and the incremental cost-effectiveness ratio (ICER).*

|  | Cost FET (€) | P correct prognosis | ICER (€) |
| --- | --- | --- | --- |
| Average | 781.1 | 0.2281 | 3,612 |
| Maximum | 846.2 | 0.4017 | 17,188 |
| Minimum | 733.4 | 0.0455 | 1,929 |
| Standard Deviation | 15.3 | 0.0489 | 959.5 |
